# Wide-band/angle Blazed Surfaces using Multiple Coupled Blazing Resonances

**DOI:** 10.1038/srep42286

**Published:** 2017-02-17

**Authors:** Mohammad Memarian, Xiaoqiang Li, Yasuo Morimoto, Tatsuo Itoh

**Affiliations:** 1 Dept. Electrical Engineering, Sharif University of Technology, Tehran, Iran; 2 Dept. Electrical Engineering, University of California Los Angeles, Los Angeles, CA, 90095, USA; 3 Mitsubishi Electric Corporation, Kamakura, 247-8501, Japan

## Abstract

Blazed gratings can reflect an oblique incident wave back in the path of incidence, unlike mirrors and metal plates that only reflect specular waves. Perfect blazing (and zero specular scattering) is a type of Wood’s anomaly that has been observed when a resonance condition occurs in the unit-cell of the blazed grating. Such elusive anomalies have been studied thus far as individual perfect blazing points. In this work, we present reflective blazed surfaces that, by design, have *multiple coupled blazing resonances* per cell. This enables an unprecedented way of tailoring the blazing operation, for widening and/or controlling of blazing bandwidth and incident angle range of operation. The surface can thus achieve blazing at multiple wavelengths, each corresponding to different incident wavenumbers. The multiple blazing resonances are combined similar to the case of coupled resonator filters, forming a blazing passband between the incident wave and the first grating order. Blazed gratings with single and multi-pole blazing passbands are fabricated and measured showing increase in the bandwidth of blazing/specular-reflection-rejection, demonstrated here at X-band for convenience. If translated to appropriate frequencies, such technique can impact various applications such as Littrow cavities and lasers, spectroscopy, radar, and frequency scanned antenna reflectors.

Blazed gratings^1^,^2^,^3^,^4^,^5^,^6^,^7^,^8^,^9^,^10^,^11^,^12^,^13^,^14^,^15^,^16^,^17^,^18^,^19^,^20^,^21^,^22^,^23^,^24^,^25^,^26^,^27^,^28^,^29^,^30^,^31^,^32^,^33^,^34^,^35^,^36^,^37^,^38^,^39^,^40^,^41^,^42^ can reflect an oblique incident wave back in the direction of incidence with low or even zero specular scattering, unlike mirrors and metal plates. The scattering of the incident wave into diffraction orders is dictated by the grating periodicity, which is in the order of the free-space wavelength for blazing. Typical blazed gratings^1^,^2^,^3^,^4^,^5^,^6^,^7^,^8^,^9^,^10^,^11^,^12^,^13^,^14^,^15^, such as the classical right-angle sawtooth grating^1^ shown in Fig. 1a, and the rectangular groove grating^2^ shown in Fig. 1b are non-planar structures that can blaze. Planar and printed gratings, such as those shown in Fig. 1c,d have also been shown to blaze efficiently^16^,^17^,^18^,^19^,^20^,^21^,^22^,^23^,^24^,^25^,^26^,^27^,^28^,^29^,^30^,^31^,^32^,^33^,^34^,^35^,^36^,^37^. Blazed gratings have been used from microwaves to optics, and in different applications such as Littrow mounts^6^ and external cavity lasers, frequency scanning antenna reflectors^38^,^39^,^40^,^41^,^42^, and Radar Cross Section (RCS) reduction surfaces^19^,^20^,^21^, to name a few.

Metasurfaces^43^, thin structures that provide control over the local phase and magnitude of reflection/transmission coefficients of the surface, can tailor the scattered wave-front. Metasurface based gratings^34^ have been employed recently to mimic the operation of blazed gratings for auto-collimation^35^,^36^. We recently demonstrated a blazed metasurface grating at microwaves in ref. 35 that mimicked the operation of a typical sawtooth right-angle grating in the Transverse Electric (TE) polarization at X-band. The unit-cell of the structure in ref. 35 is shown in Fig. 1c with its period equal to the period of standard blazed gratings such as the sawtooth grating. This blazed metasurface can scatter the incident wave back in the direction of the incidence, similar to their non-planar counterparts. The incident wave experiences an overall 2π phase modulation of the local reflection coefficient over one period. The unit-cell is made of several subwavelength regions as shown in Fig. 1c, where each region is realized with fine metal patterning to provide a particular reflection phase.

The most widely used type of blazed grating only scatters the specular (*m* = *0*) and the (*m* = −*1*) diffraction orders, while all other diffraction orders are evanescent waves. In auto-collimation condition (Littrow mount), the angle of the incident wave and the *m* = −*1* diffraction order are equal and the specular (*m* = *0*) reflection is minimized, thus most of the incident power is reflected back exactly in the path of incidence. In an infinitely periodic blazed grating such as the one shown in Fig. 1e, the amount of power scattered into each diffraction order (diffraction efficiency) is solely dictated by the cell design. A significant body of literature has studied grating diffraction efficiency^1^,^2^,^3^,^4^,^5^,^6^,^7^,^8^,^9^,^10^,^11^,^12^,^13^,^14^,^15^,^16^,^17^,^18^,^19^,^20^,^21^,^22^,^23^,^24^,^25^,^26^,^27^,^28^,^29^,^30^,^31^,^32^,^33^,^34^,^35^,^36^,^37^. For example, the right-angle sawtooth grating^1^ is only capable of modest levels of blazing in TE polarization. In other words, some amount of power always scatters into the specular order, and thus the wave is not fully reflected back on its path of incidence.

Perfect blazing (100% of incident power diffracted to the *m* = −*1* order and zero specular scattering) has been demonstrated using rectangular groove based gratings^2^,^3^ shown in Fig. 1b. At a particular depth and width of the grooves, the grating is capable of achieving 100% efficiency depending on the polarization. Moreover, it is shown that at certain dimensions, simultaneous TE and Transverse Magnetic (TM) polarization blazing can be achieved^2^,^3^. Various other grating geometries have been studied in electromagnetics and optics for their blazing effect in the past. Inspired from works on blazing of groove gratings^2^,^3^, ref. 16 demonstrated that periodic strips on a conductor backed dielectric sheet can also blaze, in effect simulating corrugated structures such as the groove grating. As another example, conductor-backed dielectric-rod based gratings were also shown to exhibit strong blazing in optics^11^. Groove gratings and strip gratings on conductor-backed dielectrics have been studied for the so-called Bragg^2^,^6^,^16^,^23^ and Off-Bragg blazing^6^,^7^,^8^,^10^,^13^,^14^,^29^,^31^ (see Methods) in both polarizations. Extending the bandwidth of finite planar blazed structures were investigated using non-periodic geometrically growing strips widths^18^, or using gratings with tapered strips in the transverse direction^21^. However, in these structures a constituent 2D unit-cell was not available which exhibited the wideband behavior.

Perfect blazing in various gratings share a common feature. The oblique incident wave couples strongly to a resonance in the unit-cell at the point of perfect blazing. For instance, at the frequency of perfect blazing in the groove grating of Fig. 1b, a resonance is formed in the grooves. Perfect blazing points are one kind of Wood’s anomalies occurring at the Bragg condition (see Methods). In explanation of Wood’s anomalies, ref. 5 described resonances supported by the structure as being responsible for such anomalies. Some anomalies were described in the context of complex leaky waves supported by the structure, where the underlying mode could be either a slow^5^ or a fast^4^ wave, and later, some anomalies were observed even when no leaky waves were supported by the structure^32^. Overall, all aforementioned Wood’s anomalies have been observed and treated as individual perfect blazing points. However, they have not yet been controllably coupled and combined towards some useful functionality or performance improvement.

The resonance perspective is an important observation for perfect blazing, and opens door for realizing various types of high-efficiency blazed gratings. In this work, we demonstrate for the first time blazed gratings comprised of a designed set of coupled blazing resonance anomalies per cell, to achieve controlled and wide blazing bands of operation at different frequencies and incident angle ranges. This is demonstrated here using coupled strips on a grounded dielectric structure under TM polarization, yielding a planar blazed surface. Other structures and polarization maybe treated in a similar coupled resonator fashion, as long as the cell geometry is appropriately designed to fit multiple resonances that couple to the incident polarization.

To this end we first study a single resonant strip^37^ in the unit-cell as shown in Fig. 1d and e. In previous studies on strip loaded grounded dielectric gratings^16^,^17^,^18^,^19^,^20^,^21^,^22^,^23^,^24^,^25^,^26^,^27^,^28^,^29^,^30^,^31^,^32^,^33^, the strip width was large (typically around half the cell size) and held constant, while other parameters such as the dielectric thickness was adjusted to observe a blazing phenomenon. In this work, we utilize a standard single layer microwave substrate with a given fixed thickness, much thinner than previous works. Blazing is achieved by the patterning of the top metallization, enabling a convenient way to tailor the blazing point at will. Moreover the lowest order strip resonance is used thus requiring the narrowest strip in the cell.

The single resonator per cell based grating is then extended to multi-resonator based gratings for the first time. Such gratings are shown to exhibit multiple perfect blazing points and a wide frequency-angle range of operation compared to existing planar and non-planar blazed gratings. The unit-cell of the blazed grating is made of several side-coupled narrow resonant strips, in a single-layer metasurface-type design. Radar Cross Section and Auto-collimation measurements show that indeed the structures can reflect significant power back in the path of incidence, over a wide frequency and angle range. It should be noted that widening the bandwidth of perfect absorbing surfaces, such as metamaterial absorbers, has been the subject of various studies in recent years^44^,^45^,^46^, utilizing double^45^ and multi^46^ resonance structures. In this work however, our goal is not to absorb the incident wave, but to provide blazing operation over a wider angle and frequency range.

The planar structures presented herein are comparable to state of the art single-layer blazed metasurface gratings^35^,^36^. Moreover, an alternative and simpler approach to metasurface blazed gratings^35^ is used here. The blazed gratings presented here only have resonant strips in the unit cell, and can blaze without the design of a phase modulated surface. Therefore their design does not require miniature metal patterning. These simple strip type patterns are not only well suited for standard microwave fabrication techniques, but may also be translated to higher frequency designs, including THz scattering surfaces based on resonant Quantum Cascade Laser waveguides^47^ implemented using microfabrication techniques.

## Results

### Single Resonator Blazed Grating

**Figure 1:**
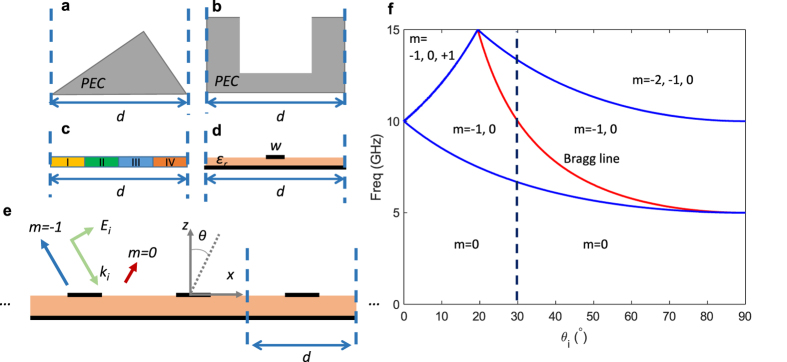
Planar and non-planar blazed gratings. Unit-cell of blazed gratings with period ‘*d*’ (**a**) right-angle sawtooth (echellette) grating, (**b**) rectangular groove grating capable of perfect blazing (**c**) Blazed metasurface^35^ realized with reflection phase regions I to IV under TE polarization, and (**d**) single resonant strip in the unit-cell^37^. (**e**) Resonant blazed grating infinitely periodic in *x* with period ‘*d*’ under Transverse Magnetic incidence, with low specular and strong *m* = −1 scatter. (**f**) Grating operation regions with different diffraction orders for various incident angles (*θ*_i_) and frequencies (*d* = 30 mm). Boundaries of region of existence of *m* = 0 and *m* = −1 orders with other regions are marked in blue. Red line shows the path of Bragg condition. Dashed vertical line shows a fixed incident angle scenario typically used in design stage.

**Figure 2:**
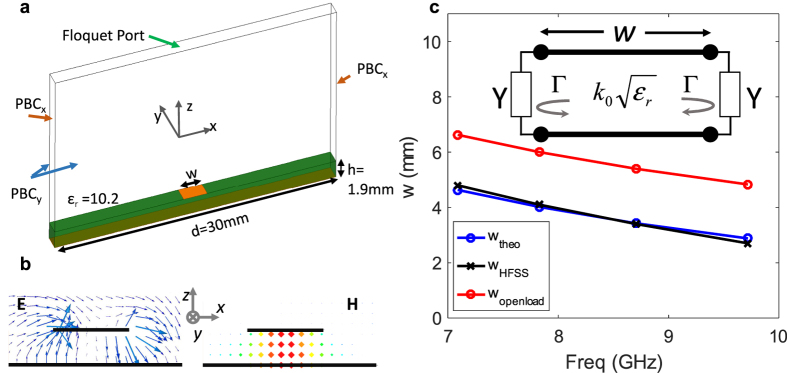
Design and modelling of the resonant strip for a desired blazing. (**a**) Unit-cell with a single resonant strip, simulated under oblique TM polarization incidence. (**b**) In-plane electric field profile as well as (**c**) the out-of-plane magnetic field profile showing resonance under the strip at perfect Bragg blazing point (*θ*_i_ = *θ*_−1_ = −30°). (**d**) Strip width required for a particular perfect Bragg blazing frequency, calculated theoretically as well as using full-wave solver.

The cross section of the unit-cell of the single resonance structure is shown in Fig. 1d and Fig. 2a. At the center of the cell, a single strip of width ‘*w*’ is situated above a grounded dielectric substrate. The vector form of the in-plane (*x-z* plane) electric field distribution near the substrate is shown in Fig. 2b, for a perfect auto-collimation (Bragg blazing) scenario. It can be seen that underneath the strip, a resonant mode is excited having two E-field maxima at the two ends of the strip with anti-parallel direction, and the H-field is out-of-plane (Fig. 2c). The field is strong underneath the resonant strip, and is much weaker in the regions of the dielectric without strip (not shown).

The fundamental quasi-TEM^y^ microstrip type mode is not excited under the strip. The TEM^y^ microstrip mode propagates in the *y*-direction without radiation. In this reciprocal situation, the incident propagating wave does not couple to the TEM^y^ mode. The incident wave couples to a higher order resonant strip mode. The field distribution of this mode is reminiscent of the higher order EH_1_ mode of a microstrip line^48^. The EH mode of the microstrip has been used in the past for leaky wave radiation^48^,^49^,^50^,^51^, when the strip is excited in the *y-*direction. In our scenario of scattering of a plane-wave, we utilize the resonant mode of the strip for scattering/re-radiation in the *x-z* plane, and no propagation of a leaky wave is exhibited in the *y*-direction.

The design of the structure for a desired perfect Bragg blazing angle and frequency is given here with simplified expressions. For auto-collimation at a desired incident angle and frequency, the period is determined by the Bragg condition (9). For example, a period of *d* = 30 mm would yield Bragg blazing of a *θ*_*i*_ = *−*30° wave at 10 GHz, which is a fairly common period used in the X-band. The cell geometry must then be chosen to yield strong blazing at the desired Bragg frequency and incident angle. For a particular substrate, the thickness and materials are fixed, and the only other available design parameter is the width of the strip. The strip width ‘*w*’ must therefore be chosen to yield resonance under the strip at the desired Bragg frequency.

A single strip on a grounded substrate, in the absence of periodicity and loading, can be modelled with the circuit shown in the inset of Fig. 2d. The circuit models the strip as a transmission line of length *w*, bouncing the Transverse Elecromagnetic wave from the two ends with reflection coefficient Γ = *e*^*jχ*^, which accounts for edge effect of the strip and the extension of the grounded substrate to its sides^52^,^53^,^54^. We can derive an expression for the desired strip width using the resonance condition underneath the strip, which results in


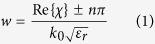


where *n* is an integer corresponding to the order of the resonant mode. The resonance order *n* = 1 is our main case of interest here as it provides the shortest strip, but higher order resonances may also be used, as long as the incident wave couples efficiently to the field distribution of the mode (typically *n* = odd in TM polarization). Equation (1) is a result of establishing the resonance condition for the circuit shown in the inset of Fig. 2d. ‘χ’ can be calculated from ref. 52 or ref. 53, for normally incident wave to the edge of the strip (wave propagation along *x* and no propagation component in the *y*-direction). In particular^53^:


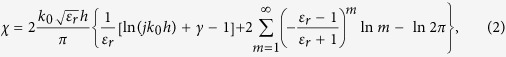


where γ = 0.5772. Alternatively, (1) can be rearranged to yield a transcendental equation in terms of frequency, in order to find the resonant frequency of a given strip width.

Figure 2d shows the required strip widths calculated using equation (1) and (2), for several Bragg frequencies of interest (blue curve). These theoretically calculated widths are in excellent agreement with the actual results obtained via the full-wave periodic unit-cell simulation under oblique incidence and Bragg condition (black curve). In the full-wave results, the frequency corresponding to the minimum of the specular reflection was found over the *m* = −1, 0 operation region of interest of Fig. 1d for each strip width. The figure also shows a third (red) curve, representing the strip widths when calculated assuming open boundaries at the two ends of the strip (*Y* = 0, or Γ = 1). Such calculation shows a large error compared to (1)–(2) and the full-wave results.

Despite the good agreement between the theoretical and full-wave results, it must be noted that utilizing this simple model for designing the strip width for a particular Bragg blazing point is based on an approximate model. The modelling ignores the coupling amount of the strip with the *m* = 0 and *m* = −1 diffraction orders and their loading effects on resonance, the mutual coupling of the two strip ends, the periodicity of the grating and its effect on resonance frequency, and it assumes the thin substrate approximation, to name a few. Nevertheless, results suggest that such effects may be considered minimal in our present scenario, and for typical substrates used in the X-band. It is also assumed that the height of the substrate is low enough to only allow for the TEM mode and no higher order modes to propagate under the strip.

**Figure 3:**
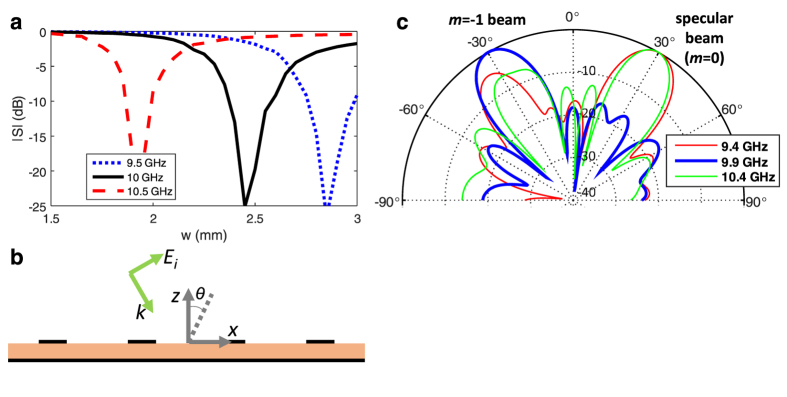
Optimization of the resonant strip. (**a**) Specular reflection as a function of strip width at three different frequencies for *θ*_i_ = −30°. A finite 4-cell sample having *w* = 2.5 mm (**b**) illuminated with a TM plane-wave. (**c**) Simulated scattering (in dB) at *θ*_i_ = −30° showing strongest blazing at 9.9 GHz from the finite sample.

The circuit model provides insight to the operation of the resonant blazed grating, and the critical parameter ‘*w*’ which is the main determinant of the blazing point. It also provides a useful expression for calculation of the strip width for the initial design. To achieve the highest amount of rejection of specular reflection, exactly at the Bragg frequency/angle of interest, the strip width can be further optimized by full-wave analysis in the periodic single-cell simulation, starting with the initial design value. Figure 3a shows the magnitude of the specular reflection collected at the top port of the cell of Fig. 2a, as a function of the strip width for three frequencies. We can see from the figure that for *θ*_*i*_ = −30° and 10 GHz operation, *w* = 2.46 mm yields the lowest specular reflection level.

The simulated scattering from a finite 4-cell sample of a grating (Fig. 3b) with *w* = 2.5 mm (chosen within the 0.1 mm accuracy level of *w*) is shown in Fig. 3c. The results show that at 9.9 GHz, the specular reflection is greatly minimized, and all power is reflected back in the *m* = −1 order. However at 9.4 GHz and 10.2 GHz, there is noticeable specular reflection. The wide lobes in the scattering pattern are due to the very finite size of the structure, and can be made narrower if higher number of periods are used, as will be shown in measurements.

Figure 3a (top)-(bottom) demonstrate the amount of specular reflection from an infinitely periodic grating, for three different strip widths of 2.7 mm, 3.4 mm, and 4.1 mm, respectively. Other parameters are unchanged, *d* = 30 mm and *h* = 1.9 mm. The color map depicts dB levels of specular reflection. It can be seen that by increasing the strip width, the very low specular reflection point (marked with a ‘X’) moves along the Bragg line to lower frequencies. This is the perfect Bragg blazing point due to the resonance effect. The change in strip width shifts the resonance frequency of the strip. Since the perfect blazing operation point always occurs on the Bragg line, it will shift along the Bragg line to a new Bragg incident angle, according to the frequency at which it resonates, following (9). At the resonant frequency of the strip, we only observe perfect blazing at the Bragg angle, and for other incident angles the specular reflection is low, but not perfectly zero.

**Figure 4:**
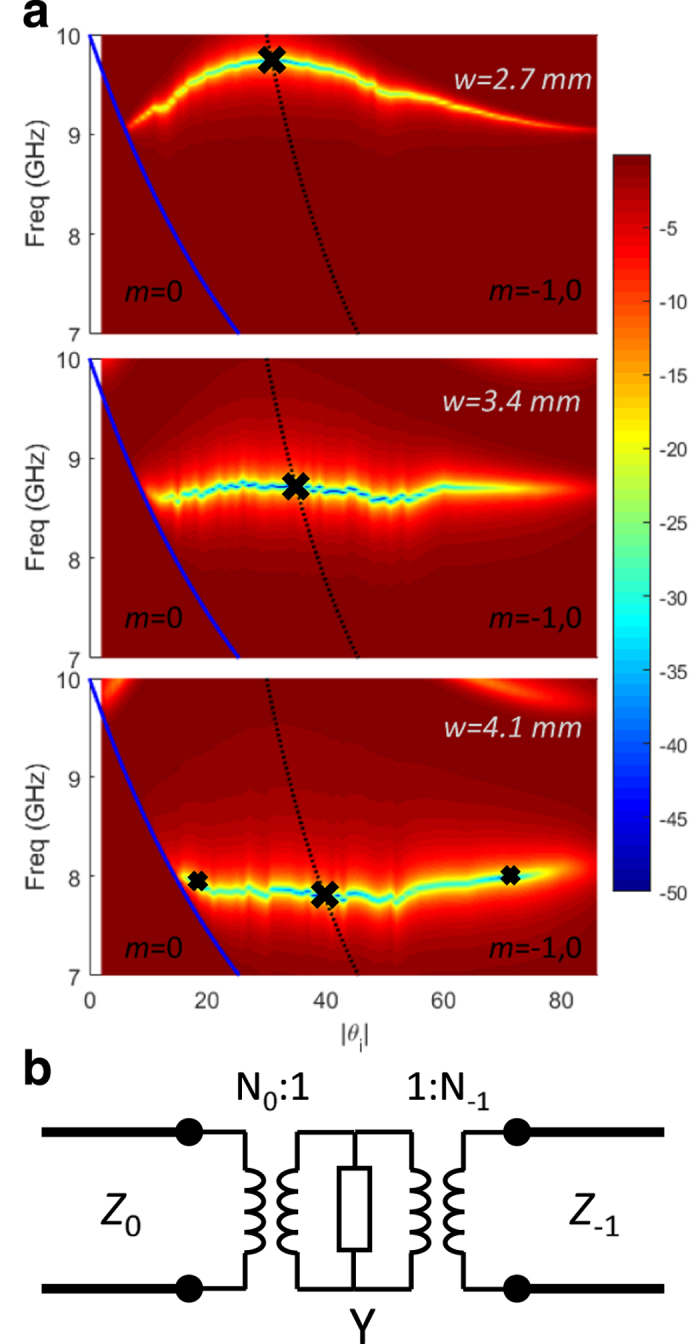
Perfect blazing points and network model. (**a**) Specular reflection for different frequency/incident angles of a unit-cell (*d* = 30 mm) having strip width, *w*, equal to (top) 2.7 mm, (middle) 3.4 mm, and (bottom) 4.1 mm. Bragg and off-Bragg blazing points marked with ‘X’ and ‘x’ respectively. (**b**) A simplified network model for reflection of the incident oblique wave to the *m* = −1 diffraction order, close to the Bragg condition. Y_eq_ lumps all higher order effects, which can essentially capture the resonance effect.

Figure 4b depicts a simplified network for the scattering of wave between the *m* = 0 and *m* = −1 (each treated as a port), for the unit cell of the grating, inspired by refs 55 and 56, but customized for the scattering of interest. Note that both ports are defined above the grating at some distance away from the surface, as was the case in Fig. 2a and all full-wave simulations.

Here the effect of all other spectral orders in the dielectric and air region are lumped in to the equivalent admittance Y_eq_(*f*). This admittance may be used to describe the resonance effect if the appropriate network is used in its stead (which would in part include the resonator described earlier). The transformers shown account for coupling of wave from each port to the equivalent circuit, which are most generally dependent on the frequency and angles of the *m* = 0 and *m* = −1 waves. The *m* = 0,−1 order TM port impedances (Z_0_ and Z_−1_) in air are


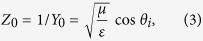



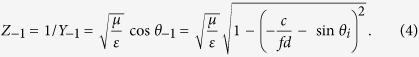


Equations (3)–(4) show that both port impedances are dependent on the incident angle, and the *m* = −1 port impedance (*Z*_−1_) is also dispersive with respect to frequency. The reflection coefficient seen for port *m* = 0 is


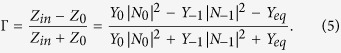


Perfect blazing, i.e. zero specular reflection and 100% efficiency into *m* = −1 is achieved at the perfect matching condition |Γ| = 0. One possible solution for such perfect matching is when the condition Y_eq_(*f*) = 0 and the condition 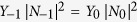 are simultaneously satisfied. Y_eq_(*f*) = 0 occurs around the internal resonance of the cell, i.e. when the sum of all spectral orders in the dielectric and air region form the appropriate resonance (which in our case is strongly related to the resonance under the strip). For the second condition however, the two impedances of (3) and (4) and the turn ratios are generally not equal, as stated earlier. However at the Bragg condition (9), the two waves are identical with respect to the grating (*θ*_i_ = *θ*_−1_) and


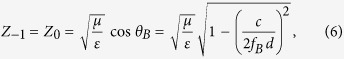



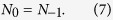


Thus when the Bragg condition 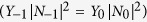 occurs at the resonance frequency of the cell (Y_eq_(*f*) = 0), specular reflection |Γ| can go to zero in (5).

It can also be seen in Fig. 4a that for the case of *w* = 4.1 mm, two additional off-Bragg blazing points exist about the Bragg line. While their nature and justification of existence is beyond the scope of this work, we note that such off-Bragg blazing phenomena are known to exist^10^,^14^,^29^,^31^, and can help to further broaden the range of angles over which specular reflection is minimized as will be shown later. Assuming a wideband model exists for the circuit of Fig. 4b, it can also be argued that these off-Bragg blazing points are other cases where |Γ| in (5) is minimized.

The Bragg and Off-Bragg blazing points used in this paper are essentially s-type Wood’s anomalies in the context of Wood’s original terminology. The observed Bragg blazing points are not Rayleigh wavelength type anomalies as they are occurring well inside the *m* = −1, 0 region. The Wood’s anomalies utilized here arise due to resonances supported by the structure (free complex resonances), as stated by the explanation of Wood’s anomalies^4^,^5^, and also verified with dispersion plots of refs 23 and 31. In this incident wave problem, we therefore tailor the forced (scattering) resonance of the structure, by choosing the width of the strip. This is a useful mechanism for tailoring the anomaly, and will be used immediately for multi-anomaly scenarios.

### Multi-resonator Blazed Grating

Given that one strip resonance enables the perfect blazing point on the Bragg line, we now investigate the effect of multiple resonances in the unit-cell for creating multiple perfect blazing points. Moreover, if these perfect blazing points are appropriately placed close to each other (appropriately coupled), a wide band/ range of frequency/incident angles may attain a low specular reflection, and a high *m* = −1 order efficiency.

Multiple resonances may be achieved in a variety of ways, for example using multi-layered structures. Here we simply utilize multiple strips placed adjacent to each other in the unit-cell to create the multiple resonance points. The strips are placed side by side with spacing, and the incident wave shines directly on all strips in the cell. This provides a simple, flat, single layer solution. To extend the single resonance concept into multiple resonances, two approaches may be taken:

#### Coupled identical resonators

**Figure 5:**
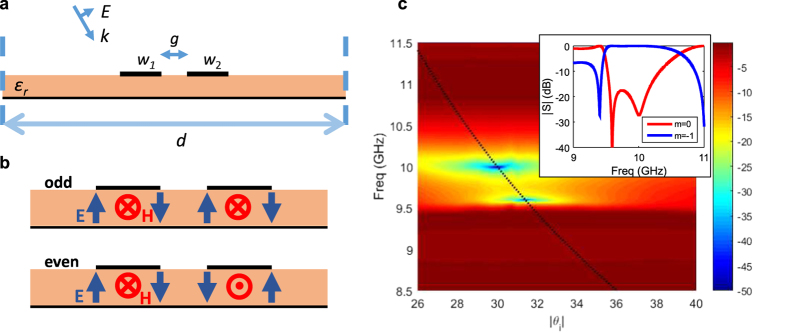
Synchronously tuned coupled blazing resonances for controlled passband. (**a**) Two coupled strips separated by a spacing within the unit-cell. (**b**) Specular reflection in dB for identical strips *w*_1_ = *w*_2_ = 2.7 mm and *g* = 4 mm. Inset shows diffraction efficiency for *θ*_i_ = −32°. (**c**) Odd and even mode profiles of the coupled resonance modes on the Bragg line.

One approach is to use two or more identical strip resonators side by side with appropriate spacing, which can act as transverse coupled resonators. An example is shown for two coupled resonators in Fig. 5a. In general, identical coupled resonators result in two new resonance frequencies based on their amount of coupling, and thus create a bandwidth of frequencies and incident angle ranges over which specular reflection is reduced. This approach, operates similar to synchronously tuned coupled resonator filters with a transverse topology^57^.

The amount of specular reflection from two coupled identical strips with *w*_1_ = *w*_2_ = 2.7 mm, and spacing *g* = 4 mm, is shown in Fig. 5b. It is evident that two new blazing resonances (the two very low specular reflection points) now appear on the Bragg line at 10 GHz and 9.595 GHz, corresponding to Bragg angles *θ*_i_ = −30° and *θ*_i_ = −31.4° respectively. It is interesting to note that the two resonances do not achieve their minimum at the same incident angle, but according to the Bragg angle. Note that the single strip of *w* = 2.7 mm has a reflection response as was shown in Fig. 4a. The two coupled strip modes are shown in Fig. 5c. The 10 GHz and 9.595 GHz coupled resonances have odd and even mode distributions with respect to the *x* = 0 plane, respectively. Under oblique incidence the symmetry of mode is slightly perturbed. Moreover the two modes couple to the incident wave unequally given their external quality factor. The odd mode has a higher amount of coupling with the incident wave, and thus provides a more pronounced region of specular reflection dip, as seen in the color plot of Fig. 5b.

The frequency response of the specular and *m* = −1 order for a fixed *θ*_i_ = −30° wave is also shown in the inset of Fig. 5b. As it can be seen, the approach results in passbands with sharp roll-offs, and with transmission zeros. The design of this two-pole passband filter response was done by optimizing the gap size between the two strips, using the single-cell periodic simulation of Fig. 2a, for the fixed *θ*_i_ = −30° incidence unit-cell simulation.

#### Coupled dissimilar resonators

In an alternative approach, two or more strip resonators of unequal and/or equal width (each with potentially a different original resonance frequency) can be placed within the unit-cell. In this approach, each resonator is potentially detuned from others, and thus targets a different range of frequencies for blazing. From a filter perspective, this is the concept of asynchronously tuned coupled resonator filters^57^.

**Figure 6:**
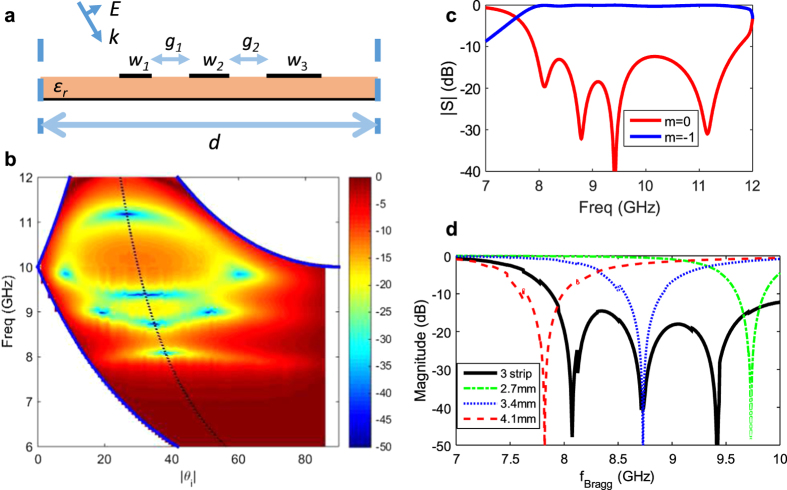
Asynchronously tuned coupled blazing resonances for wideband operation. (**a**) Three coupled dissimilar strips resonators in the blazed grating unit-cell (**b**) Specular reflection (color in dB) as a function of the incident angle and frequency, showing multiple blazing points. Dashed line shows Bragg line with multiple resonance points along it. (**c**) Specular and *m* = −1 magnitude vs frequency along the *θ*_*i*_ = 30° line. (**d**) Specular reflection (in dB) along the Bragg line for the three coupled strip design as well as each individual resonators of (**a**).

Figure 6a depicts a multi-resonance 3-strip unit-cell with strip widths *w*_1_ = 2.7 mm, *w*_2_ = 3.4 mm, *w*_3_ = 4.1 mm. The strip widths were chosen with the same linear progression of 0.7 mm, mainly for simplicity and utilizing the three Bragg resonances studied earlier in Fig. 4a. The strips are separated equally by gaps *g*_1_ = *g*_2_ = 3.5 mm. The strips are coupled to their neighbor strips within the cell, by a coupling amount determined mainly by the spacing *g*_j_. For this case, equal gap sizes were chosen, but this is not necessary and can in fact be made use to realize different coupling values for a particular frequency response. The details of the effect of gap on the coupling and frequency separation of the resonances are not discussed in this paper, but all design parameters *w*_j_ and *g*_j_ can affect the frequency response of interest. Additional discussions on this topic will be left for a future communication.

Figure 6b depicts the high level of specular reflection rejection over the wide incident angle/frequency range of interest. Multiple dips in the specular reflection plot can now be observed, and the specular reflection is observed to be low (less than −15 dB) over a wide range. See Supplementary Figure 1a for the 3D plot of this response, as well as the corresponding wideband/angle high efficiency *m* = −1 blazing response in Supplementary Figure 1b and c.

A combination of Bragg and off-Bragg high-specular-rejection points help to develop the wide angle/frequency region of specular reflection rejection. This includes several poles along the Bragg line, as well as two pairs of poles that exist symmetrically about the Bragg line. This grating thus combines multiple blazing points, to extend the range of efficient blazing. In other words it utilizes several Wood anomalies at different frequencies. Such a result is unprecedented, as typically one would achieve a particular Bragg blazing point/Wood anomaly as a consequence of a geometry. Here however, we have engineered the anomalies by choice, and placed several of such specular reflection poles in adjacent to each other to create a desired passband as shown in Fig. 6c. The figure also shows a fourth dip in the specular reflection at higher frequencies, which corresponds to a higher frequency resonance in the cell (not described herein), and is helping to even further extend the passband region.

Figure 6d depicts the amount of specular reflection from such grating extracted along the Bragg line of Fig. 6b. The figure also super-imposes the Bragg line frequency response of the three individual strips, extracted from the three cases of Fig. 4a, showing how the three individual asynchronous Bragg resonances mix to develop the multi-resonance response.

Utilizing multiple side-coupled resonances in the unit-cell for controlling multiple blazing points (be it with non-planar or planar type structures) has not yet been demonstrated as it is difficult to achieve in many blazed grating structures. This is primarily due to the types of geometries used in blazed gratings, such as wide grooves^2^,^3^ or wide strips^16^. In such gratings, there typically could only be one resonator fitted in the unit-cell. Here, we not only make the structure planar and low profile, but realize multiple high blazing efficiency points by utilizing narrow compact strip resonators in the unit-cell. The coupled resonance designs here were performed using the single-cell periodic simulations described earlier, with *θ*_*i*_ = −30°. Optimization on the gap coupling was performed to achieve the desired passband behavior. A rigorous treatment of the response in terms of filter theory can be considered as future work.

Multi-resonance Frequency Selective Surfaces FSSs have been known which operate similar to coupled resonator filters. FSSs however operate based on tailoring the reflection/transmission frequency response of the reflected/transmitted *m* = 0 ‘specular’ wave. Thus FSSs are normally operated at periods below *λ*_*0*_*/2* to avoid grating orders. In fact grating orders are considered undesirable in FSS design. Our objective however, is to apply the bandpass frequency response for transmission between the incident wave (port 1) and the *m* = −1 grating order (port 2), for a particular polarization. Thus the multi-resonance approach was applied here to a planar FSS based blazed grating, i.e. FSSs that are operating with one order grating order, and tailor the frequency response of a reflective FSS grating, from the zero-th order to the first grating order.

### Measurements at X-Band

#### Single resonance blazed grating

**Figure 7:**
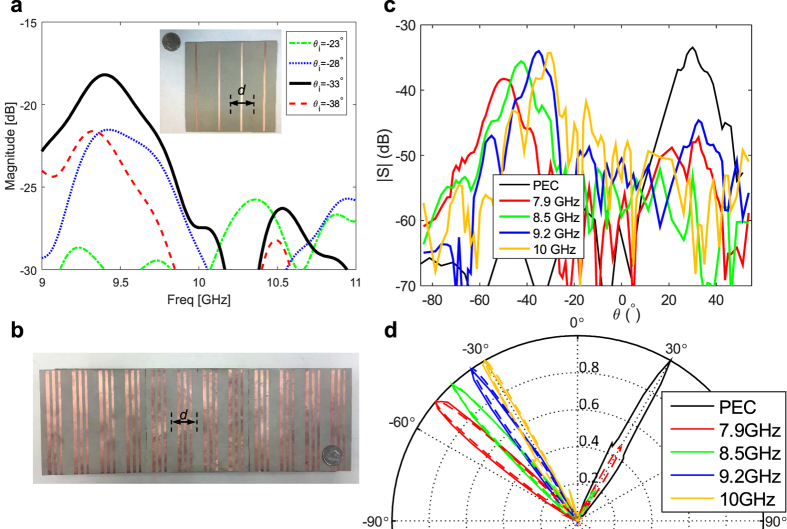
Measurements of wide angle and wideband blazing. (**a**) Auto-collimation measurements of the single resonance blazed grating in TM polarization, using the monostatic radar measurement setup^37^. Inset shows the 4-cell sample that is measured. (**b**) The 12-cell long 3-strip/cell strip blazed grating. (**c**) Measured scattered field from grating with a fixed incident beam at *θ*_i_ = −30°: for different frequencies showing wide-band rejection of specular waves and strong blazing (**d**) measured (thick dashed lines) and simulation (thin solid lines) of the normalized radiation pattern, showing agreement and frequency scanning of the blazing beam.

A 4-cell (12 cm by 12 cm) sample of the single strip blazed grating of *w* = 2.5 mm was fabricated as shown in the inset of Fig. 7a, containing 4 grating cells i.e. 4 transverse resonant strips. Figure 7a shows the results of the auto-collimation measurements, for several incident angles. While the absolute magnitude of the vertical axis in the figure is not to be interpreted, the relative magnitude of the different curves are of importance. It can be seen that at 9.4 GHz, the received reflected power by the horn is highest, for the incident angle of *θ*_*i*_ = −33°. This is in close agreement with the auto-collimation angle of *θ*_*i*_ = −32.13°, for a *d* = 30 mm grating operating at 9.4 GHz. Not all measured incident angles are shown in the figure for clarity, and only the highest (*θ*_*i*_ = −33°) and three neighboring incident angles are shown. However, other incident angles resulted in lower amounts of auto-collimation over the measured frequency band, as expected.

Additionally, we observed that the level of auto-collimation achieved at *θ*_*i*_ = −33° is comparable to that from a conducting copper sheet of the same size at normal incidence. This is an indication that the amount of auto-collimation reflection is very strong and the device is working in a near perfect auto-collimation regime, due to the resonant strips.

The finite and relatively small board size of the blazed grating, tolerances in the strips’ spacing and widths, slight variations in the dielectric constant of the substrate, all contribute to the difference in peak auto-collimation frequency between measurements and simulations.

#### Multi-resonance blazed grating

A 12-cell (36 cm by 12 cm large) sample was fabricated based on the design parameters of the 3-strip grating in Fig. 6, and bistatic radar measurements were performed on the sample. The fabricated sample is shown in Fig. 7b. Aside from the 12-cell multi-strip sample, a reference PEC (Perfect Electric Conductor) plate of the same size was also measured for all angles.

The radiation pattern of the scattered field from both the sample and the PEC plate are depicted in Fig. 7c–d at different frequencies. The incident beam in this bistatic case was fixed at *θ*_i_ = −30°. It can be seen that the copper plate has complete specular reflection with a strong beam at *θ*_s_ = + 30°. The multi-resonance blazed grating on the other hand has a very low specular reflection, and a strong backscatter beam pointing at *θ*_s_ = −30° at 10 GHz. The amount of specular reflection from the blazed grating is always more than 10 dB lower than specular reflection of the copper plate, over the measured bandwidth.

The backscatter *m* = −1 beam also exhibits a frequency scanning behavior. As the frequency is decreased from 10 GHz to 7.9 GHz, the *m* = −*1* beam scans from *θ*_s_ = −30° towards *θ*_s_ = −50°. This can be seen in the plots of Fig. 7c and the normalized radiation pattern (|E_normalized_|^2^) in Fig. 7d. The simulated results are also in good agreement with the measured results, and are super-imposed in Fig. 7d. The experimental results show higher level of specular rejection for lower frequencies than the simulation results, due to losses and fabrication tolerances. Measurement results for above 10 GHz were not reported due to blocking of the main beam by the receive and transmit horns in our bistatic radar setup. However the grating is expected to have high *m* = −1 efficiency even above 10 GHz, due to higher order Bragg and Off-Bragg perfect blazing points observed in Fig. 6b. As expected, the scattered *m* = −1 beamwidth broadens at lower frequencies. This is due to the reduction of the effective aperture area of the grating for more oblique radiation as the frequency is scanned, thus reducing the peak gain and broadening of the beam. Such wideband frequency scanning backscattered beams are known to have applications in frequency scanned reflector antennas^38^,^39^,^40^,^41^,^42^.

## Discussion

Planar blazed gratings with multiple coupled resonances within the unit-cell can result in multiple perfect blazing points in the Bragg and off-Bragg conditions (multiple coupled Wood anomalies). When designed appropriately, these multiple resonances can extend the bandwidth/incident angle range of blazing similar to bandpass filters. Prototypes were experimentally demonstrated at X-band, showing feasibility of designs using standard microwave fabrication. When scaled to appropriate frequencies, these gratings can find important applications in lasers, radar and stealth, and frequency scanned antennas, to name a few.

## Methods

### Diffraction and perfect blazing

The period of any blazed grating (be it non-planar or planar/metasurface based) is what ultimately determines the diffraction angles. The governing equation of operation of a grating^58^ for propagating waves in vacuum relates the periodicity of the structure (*d*) along one dimension, the diffracted order (*m*), the operating wavelength (*λ*_*0*_), the angle of incidence (*θ*_*i*_), and the angle of the diffracted order (*θ*_*m*_) by:


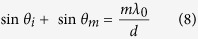


where angles are measured with respect to the *z*-axis as shown in Fig. 1e. The blazed gratings of interest here are ones operating in the region where only the *m* = 0 (specular) and the *m* = −*1* orders simultaneously propagate, and all other orders are evanescent. For a particular grating period, the incident angle and frequency of operation determine this region.

When the grating is used in the Littrow mount (a.k.a. auto-collimation), the incident and the scattered *m* = −*1* wave angles are identical (*θ*_*i*_ = *θ*_−*1*_), thus the periodic structure can reflect the incident wave back in the direction of incidence (valid for *1/3* <sin*θ*_*i*_*|< 1* to not allow other diffraction orders). It simply follows from (8) that for such auto-collimation to occur, the Bragg condition must be satisfied^2^:


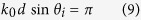


While a period satisfying (9) guarantees the *m* = *−1* diffraction angle to be exactly in the incident direction, it does not guarantee perfect auto-collimation (i.e. zero *m* = 0 specular reflection and all power diffracted into the *m* = −1 order). Thus the scattered wave is typically a combination of both the *m* = 0 and *m* = −1 scattered orders. The design/geometry of the unit-cell determines how the scattered power is distributed between the *m* = *0* and *m* = −*1* orders.

Figure 1f shows the diffraction operation regions of a grating with *d* = 30 mm, having auto-collimation at 10 GHz for *θ*_*i*_ = *θ*_−*1*_ = −30°. The region of interest in our work is where the *m* = 0,−1 only exist, as shown. The region boundaries (blue traces) are simply found from (8)^14^. The Bragg line is also depicted in the figure. The Bragg line (red trace) is essentially (9), which is the operation path where auto-collimation is achieved.

In the *m* = −1, 0 region of interest in an infinitely long lossless blazed grating, the sum of power scattered into the specular and the *m* = −1 order equal to the incident power. Thus in many cases discussed in this paper, we investigate the amount of specular reflection from the grating to go to zero, which is in turn is an indication of high amount of *m* = −1 efficiency.

Figure 1f also shows a dashed line along the *θ*_*i*_ = 30°. In many of our simulations, we utilize this scenario, and observe the frequency response. Such a scenario is where the incident beam is fixed at *θ*_*i*_, and frequency varies in the *m* = −1, 0 region. In such circumstances, the oblique incident beam is fixed, but the *m* = −1 beam moves in one direction according to (8).

### Structure and Simulations

The period of the blazing structures used here, *‘d’*, is chosen as 30 mm in order to satisfy the Bragg condition in the X-band. A grounded Rogers Duroid 6010 substrate is used with dielectric constant of 10.2, dielectric loss tangent of 0.002, substrate height *h* = 1.9 mm and conductor conductivity of 5.85 × 10^7^ S/m, similar to a typical microwave substrate used for metasurface blazed grating^35^.

Figure 2a shows the unit-cell used for the simulation of the grating. Periodic single cell simulations show the operation of the structure. The simulation uses periodic boundary conditions (PBC) with a phase difference of *k*_*0*_*d*sin(*θ*_*i*_) in the *x*-direction. The structure is illuminated from the top Floquet port supporting the oblique incident wave, and the specular *m* = 0 and *m* = −1 reflections are collected at this top port. The *y*-dimension of the simulated unit-cell is chosen as very narrow (1.5 mm) and a *y*-direction periodic boundary condition with phase shift of 0° is used to essentially simulate a complete 2D simulation in the *x-z* plane. The simulations were performed using Ansoft HFSS.

Initial design stage simulations were first performed with lossless materials, such that only specular reflection minima was needed to search for the resonance frequency. Simulations were then repeated with all losses included, which showed minimal effect on the *m* = −1 back reflected wave at these frequencies. Final measured results were also compared to simulation results which included all losses. Such single-cell simulations with losses can be a useful tool especially at higher frequencies including THz^59^, to provide an estimate of the loss effects on the amount of *m* = −1 reflected power.

### Measurements and Experimental Setup

The single resonator blazed grating, and the three strip per cell multi-resonator blazed grating were fabricated for demonstration purposes, and scattering measurements were performed. The identical two-strip example was not demonstrated experimentally, as a narrow band result was expected from simulations. The single resonator sample was measured only for auto-collimation, whereas the multi-resonance blazed grating was measured for bistatic scattering scenario, for applications in frequency scannable reflector antennas.

For auto-collimation measurements, the procedure and setup outlined in ref. 37 was used. A standard X-band horn antenna was used to shine linearly polarized waves onto the samples. The sample is placed on a rotator which measures the angle from the reference zero. The sample is placed farther than the far-field limit of the horn specified by 2*D*^*2*^*/λ*_*0*_, where *D* is the diagonal of the horn cross-section at the lowest operating wavelength. The horn antenna is connected to the vector network analyzer to perform single port measurement, which is the amount of reflected power from the environment. When shining on the sample, this setup essentially always measures the amount of *m* = −1 reflection for *θ*_*i*_ = *θ*_−*1*_, i.e. the strength of auto-collimation. In order to rule out effects of slight mismatch at the input of the horn, the measured S-parameters were time-gated. Measurements of each sample were performed at intervals of 5°.

For characterization of the specular and back scattering from the multi-resonance grating, a bistatic radar cross-section setup was established. Supplementary Figure 2 shows the diagram of the setup, utilizing two X-band horn antennas for the transmit and receive signals, and a Vector Network Analyzer (VNA). The horns were fed using coaxial cables and a coaxial to waveguide transition was used at the input of each horn. The incident beam was fixed at *θ*_i_ = 30° shining onto the sample, while the receive horn rotated about the sample over all angles to capture the scattered pattern. An isolator was also used at the input of the receive horn, to avoid any calibration drift over time due to the long cable used for the rotating receive horn. The captured raw frequency-domain S-parameters was inverse Fourier transformed to time-domain, then time-gated to remove an initial pulse due to direct horn-to-horn coupling, and then Fourier transformed back into frequency domain.

## Additional Information

**How to cite this article**: Memarian, M. *et al*. Wide-band/angle Blazed Surfaces using Multiple Coupled Blazing Resonances. *Sci. Rep.* **7**, 42286; doi: 10.1038/srep42286 (2017).

**Publisher's note:** Springer Nature remains neutral with regard to jurisdictional claims in published maps and institutional affiliations.

## Supplementary Material

Supplementary Information
