# Supplementary material for: Wide-band/angle Blazed Surfaces using Multiple Coupled Blazing Resonances
Source: Sci Rep. 2017 Feb 17;7:42286. doi: 10.1038/srep42286 (PMC5314452; doi:10.1038/srep42286)
Supplement: Supplementary Information [file srep42286-s1.doc]

Wide-band/angle Blazed Surfaces using Multiple Coupled Blazing Resonances

Mohammad Memarian^1^*, Xiaoqiang Li^2^, Yasuo Morimoto^3^, Tatsuo Itoh^2^

1. Dept. Electrical Engineering, Sharif University of Technology, Tehran, Iran.

2. Dept. Electrical Engineering, University of California Los Angeles, Los Angeles, CA, 90095, USA.

3. Mitsubishi Electric Corporation, Kamakura, 247-8501, Japan.

*Corresponding author: [mmemarian@sharif.edu](mailto:mmemarian@sharif.edu)

Supplementary Information


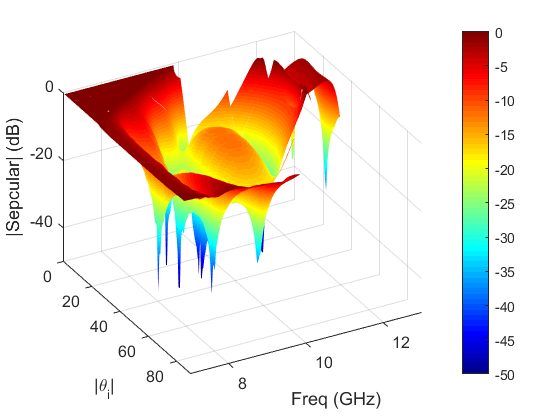


(a)


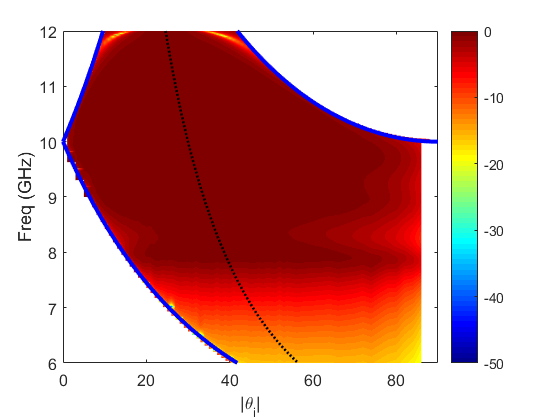


(b)


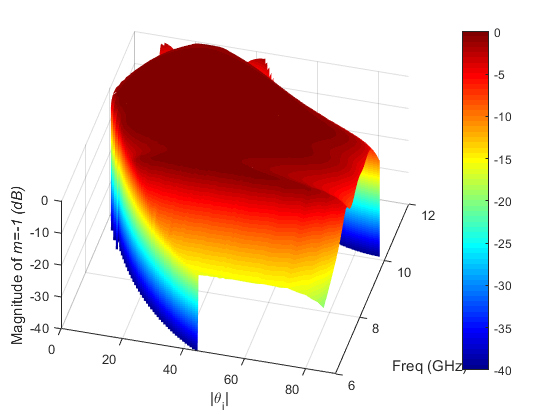


(c)

(a) 3D view of the wide angle/band reduction of specular reflection from the multi-resonance (3 strip per cell) blazed grating of Fig 6 in manuscript, and the corresponding (b) Wide angle/band high-efficiency blazing of *m*=-1 mode in dB (c) 3D view of (b).


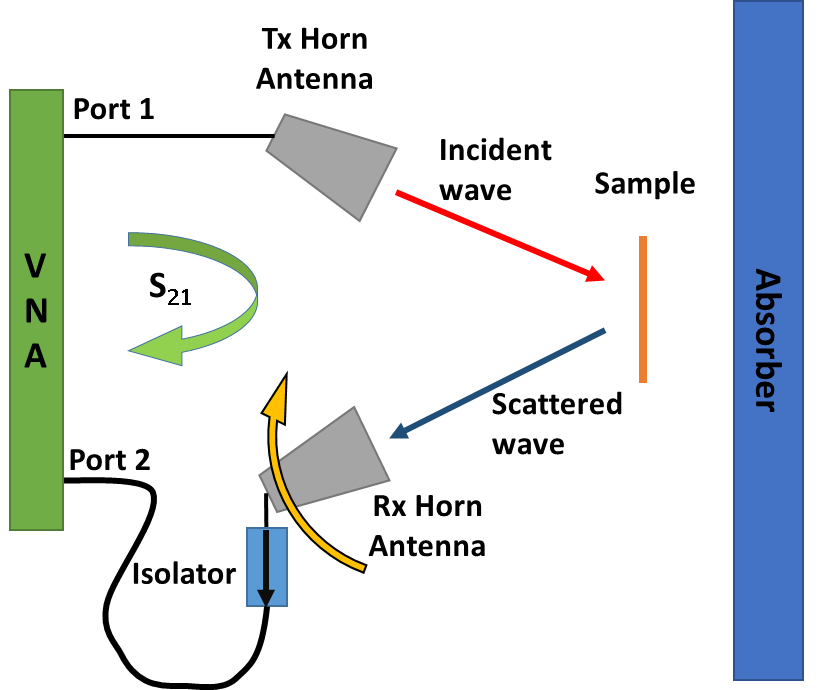


Bistatic radar measurement setup.
